# DKK3 blocked translocation of β‐catenin/EMT induced by hypoxia and improved gemcitabine therapeutic effect in pancreatic cancer Bxpc‐3 cell

**DOI:** 10.1111/jcmm.12675

**Published:** 2015-09-23

**Authors:** Qingqu Guo, Wenjie Qin

**Affiliations:** ^1^ Department of SurgerySecond Affiliated HospitalCollege of MedicineZhejiang UniversityHangzhouChina

**Keywords:** DKK‐3, β‐catenin, EMT, pancreatic cancer, chemotherapy

## Abstract

The Wnt/β‐catenin signalling pathway is activated in pancreatic cancer initiation and progression. Dickkopf‐related protein 3 (DKK3) is a member of the human Dickkopf family and an antagonist of Wnt ligand activity. However, the function of DKK3 in this pathway in pancreatic cancer is rarely known. We examined the expression of DKK3 in six human pancreatic cancer cell lines, 75 pancreatic cancer and 75 adjacent non‐cancerous tissues. Dickkopf‐related protein 3 was frequently silenced and methylation in pancreatic cancer cell lines (3/6). The expression of DKK3 was significantly lower in pancreatic cancer tissues than in adjacent normal pancreas tissues. Further, ectopic expression of DKK3 inhibits nuclear translocation of β‐catenin induced by hypoxia in pancreatic cancer Bxpc‐3 cell. The forced expression of DKK3 markedly suppressed migration and the stem cell‐like phenotype of pancreatic cancer Bxpc‐3 cell in hypoxic conditions through reversing epithelial–mesenchymal transition (EMT). The stable expression of DKK3 sensitizes pancreatic cancer Bxpc‐3 cell to gemcitabine, delays tumour growth and augments gemcitabine therapeutic effect in pancreatic cancer xenotransplantation model. Thus, we conclude from our finding that DKK3 is a tumour suppressor and improved gemcitabine therapeutic effect through inducing apoptosis and regulating β‐catenin/EMT signalling in pancreatic cancer Bxpc‐3 cell.

Pancreatic cancer has long been well known as one of the aggressive malignant disease with worst prognosis 1. The poor prognosis of pancreatic cancer is attributable to its poor response to chemotherapy, aggressive local invasion and early metastases 2. Presently, gemcitabine has become the standard therapy for advanced pancreatic cancer. However, gemcitabine alone is not very effective and is associated with drug resistance 3. In view of the problem of gemcitabine drug resistance, developing new agents and innovative approaches are the continuing efforts of research to advance the treatment 4.

Epithelial–mesenchymal transition (EMT) is a process by which epithelial cells lose epithelial characteristics and gain a mesenchymal phenotype, as characterized by down‐regulation of E‐cadherin and up‐regulation of mesenchymal molecular markers such as vimentin 5. One of the signals initiating EMT is Wnt pathway whose stimulation triggers nuclear translocation of β‐catenin, which in turn forms a complex with T cell factor(TCF) transcription factors to transcribe target genes, resulting in activation of c‐myc and cyclinD1 6. Furthermore, a strong correlation between EMT and drug resistance has been established 7. Research results have confirmed that the EMT cells were a resource for cancer stem‐like cells which are more resistant to therapies, because of the survival advantage with increased anti‐apoptotic activities 8.

Dickkopf‐related protein 3 (DKK3) is a member of the human Dickkopf family encoding secreted proteins that control cell fate during embryonic development 9. The DKK3 protein is ubiquitously expressed in normal human tissues, but this molecule was subsequently recognized to display remarkably decreased expression in several cancers 10, 11, 12. Thus, DKK3 was a potential tumour suppressor. In contrast to other family members, DKK3 is a divergent member with regard to biological functions. Furthermore, whether antagonize 13 or enhance 14 Wnt/β‐catenin signalling is controversial from previous reports.

However, till date, there is rare information as to the expression of DKK3 and its function in EMT and chemotherapy in pancreatic cancer. In this study, we assessed the expression of DKK3 in pancreatic cancer specimens. We also investigated its relationship with EMT and chemotherapy in pancreatic cancer. Our results demonstrated that DKK3 regulated EMT and improved gemcitabine therapeutic effect in pancreatic cancer.

## Materials and methods

### Cell culture and hypoxia procedure

The human pancreatic cancer cell lines Aspc‐1, Bxpc‐3, CFPAC‐1, MiaPaCa‐2, PANC‐1 and SW1900 were purchased from American Type Culture Collection (ATCC, Rockville, MD, USA). MiaPaCa‐2, Panc‐1 were maintained in DMEM supplemented with 10% foetal bovine serum, 100 units/ml penicillin and 100 mg/ml streptomycin in a humidified incubator containing 5% CO_2_ in air at 37°C. Aspc‐1, Bxpc‐3, CFPAC‐1 and SW1900 were maintained in RPMI 1640 containing supplements as above. For all experiments, the cell lines were treated in serum‐free medium (0.1% bovine serum albumin, 5 mg/ml transferrin and 5 ng/ml sodium selenite and antibiotics). Hypoxic conditions (1% O_2_) were created with a three‐gas incubator MiniGalaxy A (RS Biotech, Irvine, UK) by injection of N2 (1% O_2_/94% N_2_/5% CO_2_ atmosphere, 37°C). In other experiments hypoxia mimetic conditions were chemically generated by treating cells with 100 mM cobalt chloride (CoCl_2_; Sigma‐Aldrich, St. Louis, MO, USA) for the indicated times.

### Reagents

CoCl_2_ was purchased from Sigma‐Aldrich. Gemcitabine was purchased from Ely Lilly (Bad Homburg, Germany) and dissolved in sterile 0.9% sodium chloride to make 50 g/l stock solution.

### Methylation‐specific PCR analysis of DKK3 promoter

Dickkopf‐related protein 3 promoter methylation status was analysed by using methylation‐specific PCR (MSP). Bisulphite‐modified DNA was amplified by two different primer pairs specific to the unmethylated (u) and methylated (m) promoter sequences respectively. The methylation‐specific primers are m3: 5′‐GGGCGGGCGGCGGGGC, m4: 5′‐ACATCTCCGCTCTACGCCCG; The unmethylation‐specific primers are u3: 5′‐TTAGGGGTGGGTGGTGGGGT, u4: 5′‐CTACATCTCCACTCTACACCCA. PCR amplification was performed for a total of 34 cycles with an annealing temperature of 65°C and 59°C respectively. Non‐methylated and methylated human DNA were used as negative and positive controls respectively. Methylation‐specific PCR products were then analysed by a 2% agarose gel containing 100 bp DNA markers (MBI Fermentas, Vilnius, Lithuania).

### Overexpression of DKK3 by lentiviral particles

Production of lentiviral particles was carried out according to the manufacturer's protocol (Addgene, Cambridge, MA, USA) using the pLV‐5 full‐length cDNA of DKK3 for overexpression. The empty pLV‐5 vector was used as normal controls. For viral transduction, cells were seeded in appropriate vessels and left to adhere overnight. Thereafter, medium was replenished and supplemented with virus‐containing supernatant at MOI 0.5 (overexpression).

### Cell transwell assays

Cell transwell assays was evaluated using Transwell migration chambers (8 μm pore size; Millipore Corporation, Billerica, MA, USA). About 100 μl cell suspension with serum‐free medium were seeded in the upper chamber (1 × 10^6^ cells/ml). The lower chamber contained 600 μl medium with 10% fetal bovine serum(FBS) as chemotaxin. After 48 hrs transfection, cells were stained with crystal violet staining solution (Beyotime, Nantong, China). Migrated cells were counted using Image‐pro Plus 6.0 (Media Cybernetics, Rockville, MD, USA) while cell numbers of the normal control group were normalized to 1. All experiments were performed in triplicate independently.

### Cell proliferation assays

Cell counting kit‐8 (CCK‐8) assay (Beyotime) was used to detect the cell proliferation after 24 hrs transfection, accompanied by the employment of the TECAN infinite M200 Multimode microplate reader (Tecan, Mechelen, Belgium) to measure the absorbance at 450 nm. All experiments were performed in triplicate independently.

### Immunofluorescence analysis

For immunofluorescence analysis, cells were plated in six‐well chamber slides for 24 hrs before being incubated with 100 μM CoCl_2_. Afterwards, cells were fixed in 4% paraformaldehyde for 10 min. at room temperature, permeabilized with 0.1% Triton X‐100 in 0.01 M PBS (pH 7.4) containing 0.2% bovine serum albumin, air‐dried and re‐hydrated in PBS. Cells were then incubated with a rabbit polyclonal antibody against active‐β‐catenin 15, diluted 1:200 in PBS containing 1% normal goat serum for 2 hrs at room temperature. Negative controls were performed by omitting the primary antibody. After three washings in PBS for 10 min., an anti‐rabbit IgG fluorescein isothiocyanate(FITC)‐conjugated secondary antibody (Santa Cruz Biotech, Santa Cruz, CA, USA) diluted 1:250 in PBS was added for 2 hrs at room temperature. Nuclei were stained with 4',6‐diamidino‐2‐phenylindole(DAPI) (Invitrogen, Rockville, MD, USA). Observation and image acquisition were performed with a confocal microscope (Zeiss, Oberkochen, Germany).

### Immunohistochemical analysis

Tissue arrays of pancreatic cancer specimen were purchased from Shanghai Outdo Biotech Co., Ltd (Shanghai, China). Sections were cut from paraffin embedded pancreatic tumour tissues. Immunostaining was performed with primary antibodies specific for DKK3 13, β‐catenin, E‐cadherin (Epitomics Inc., Burlingame, CA, USA) 16 and CD133 (Santa Cruz Biotech) with appropriate dilutions and using normal host serum for negative controls, followed by staining with appropriate horse radish peroxidase(HRP)‐conjugated secondary antibodies. The slides were developed in diaminobenzidine and counterstained with a weak solution of haematoxylin solution stain. The stained slides were dehydrated and mounted in permount and visualized on an Olympus microscope (Olympus, Shinjuku, Tokyo, Japan).

### Flow cytometric assessment of apoptosis

The measurement of phosphatidylserine redistribution in a plasma membrane was conducted according to the protocol outlined by the manufacturer of the Annexin V‐FITC/PI apoptosis detection kit (Abcam, Cambridge, MA, USA). The stained cells were analysed directly by flow cytometry using the Cell Quest program (Becton Dickinson, San Jose, CA, USA).

### Western blot analysis

Whole‐cell lysates were prepared using radio immunoprecipitation assay(RIPA) lysis buffer (1% Triton X‐100, 1 mmol/l ethylenediaminetetraacetic acid, 100 mmol/l NaF, 1 mmol/l Na_3_VO_4_ and protease inhibitor cocktail), and Western blot analyses were performed as described previously 17. The following antibodies were used: β‐catenin, DKK3, cyclin D1, c‐Myc (Santa Cruz Biotech) 18, E‐Cadherin (Epitomics Inc.), vimentin, cleaved caspase‐3, cleaved caspase‐9, cleaved caspase‐7, cleaved PARP, E‐cadherin, vimentin, N‐Cadherin, Histone 1 (Cell Signaling Technology, Danvers, MA, USA) 19, β‐actin (Sigma‐Aldrich) 20, survivin, OCT4 and CD133 (Santa Cruz Biotech) 21, Glyceraldehyde 3‐phosphate dehydrogenase (Abcam) 22.

### Animals study

Around 4–6‐week‐old male athymic nu/nu mice were obtained (Zhejiang Chinese Medical University) for tumour implantation. About 7 × 10^6^ Bxpc‐3 cells were injected subcutaneously in the backs of 20 g athymic nu/nu mice. Once tumour masses became established and palpable, animals were randomized to receive intraperitoneal injections of vehicle (0.9% sodium chloride), gemcitabine (125 mg/kg) 23 twice per week for 2 weeks. Tumour volumes and bodyweight were measured weekly. Tumour volume was measured along the longest orthogonal axes and calculated using the formula: Volume = length × width^2^/2, where width was the shortest measurement. At the end of the experiment, subcutaneous xenografts were excised and stocked in 4% formaldehyde and embedded in paraffin.

### Statistical analysis

Data were expressed as mean values ± S.E. and analysed by a two‐tailed *t*‐test with *P* < 0.05 considered significant. Non‐parametric data were compared by the chi‐squared test or Fisher's exact test. Analyses were performed with SPSS 15.0 statistical software package (SPSS Inc., Chicago, IL, USA).

## Results

### Expression of DKK3 and DKK3 methylation in pancreatic cancer cells

**Figure 1:**
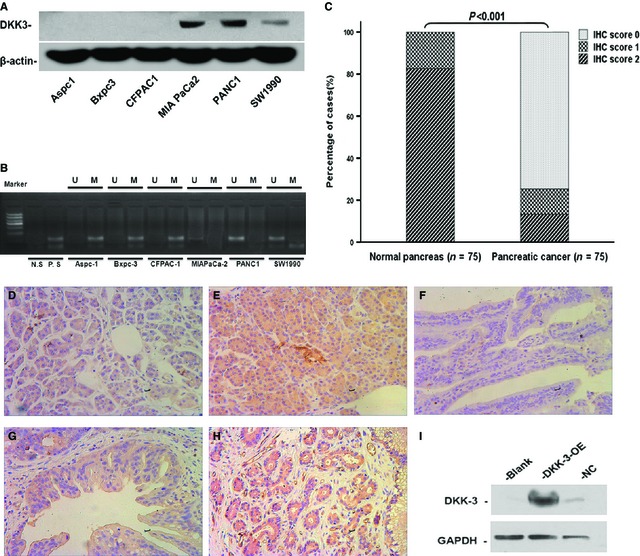
Expression of Dickkopf‐related protein 3 (DKK3) in pancreatic cancer cell lines and pancreatic cancer tissues. (**A**) Expression of DKK3 in pancreatic cancer cell lines detected by Western blot, with β‐actin as a control. (**B**) Methylation status of DKK3 in pancreatic cancer cell lines. M: methylated; U: unmethylated. (**C**) Summary of DKK3 expression in human normal pancreas and pancreatic cancer samples. Representative immunostaining of DKK‐3 in human normal pancreas, immunohistochemical (IHC) score 1 (**D**), 2 (**E**) and pancreatic cancer samples, IHC score 0 (**F**), 1 (**G**) and 2 (**H**). (**I**) Overexpression of the DKK3 gene was confirmed by Western blotting in Bxpc‐3 Cells.

To determine the expression of DKK3, we detected the DKK3 level in six human pancreatic cancer cell lines (Aspc‐1, Bxpc‐3, CFPAC‐1, MiaPaCa‐2, PANC‐1 and SW1900). As shown in Figure 1A, DKK3 expression was significantly higher in MiaPaCa‐2, PANC‐1 cells, followed is SW1900 cells. Dickkopf‐related protein 3 was not expressed in Aspc‐1, Bxpc‐3, and CFPAC‐1 cells. Because promoter methylation was a reason for silencing the DKK genes in human cancer, so we further investigated the methylation status of the DKK3 promoters. Methylation‐specific PCR analysis revealed that DKK3 methylation were detected in pancreatic cancer cell lines Aspc‐1, Bxpc‐3, CFPAC‐1, which were in agreement with the result of Western blotting (Fig. 1B). Next, the expression levels of DKK3 protein in 75 pancreatic cancer and adjacent non‐cancerous tissues were examined by immunohistochemical (IHC) analysis. Clinicopathological features of the patients included in this study are summarized in Table 1. Typical IHC findings of DKK3 in pancreatic cancer and non‐cancerous tissue specimens were shown in Figure 1D–H. The expression of DKK3 was significantly lower in pancreatic cancer tissues than in adjacent normal pancreas tissues (Fig. 1C).

**Table 1:** Clinicopathological characteristics of 75 pancreatic cancers

Clinicopathological features	Numbers (*n* = 75)
Age at diagnosis (years)	
<60	31
≥60	44
Sex	
Male	48
Female	27
Tumour location	
Head	55
Body and tail	20
Perineural invasion	
No	43
Yes	32
pTNM stage	
I	43
II	28
III	0
IV	4
Tumour size	
≤4 cm	46
>4 cm	29
Lymphatic metastasis	
Negative	49
Positive	26
Distant metastasis	
Negative	71
Positive	4
Pathological grading	
I	3
II	53
III	19

### Overexpression of DKK3 inhibits nuclear translocation of β‐catenin induced by hypoxia in pancreatic cancer Bxpc‐3 cell

For this study, pancreatic ductal carcinoma cell lines Bxpc‐3 were transfected with a DKK3 expression construct. In contrast to the normal control groups (NC group, vector‐transfected), the levels of DKK3 protein secreted into the culture media were greater for DKK3 overexpression (DKK3‐OE) group cells (Fig. 1I).

β‐catenin is a key component of the Wnt/β‐catenin signalling pathway. The accumulated β‐catenin promotes its translocation to the nucleus and interacts with TCF transcription factors to modulate the transcription of downstream target genes. β‐catenin is activated in hypoxic conditions 24. In addition, oxygenation measured intraoperatively with an electrode shows high levels of hypoxia in pancreatic cancers 25. Meanwhile, it is reported that β‐catenin/Tcf/Lef pathway is frequently altered in pancreatic cancers 26 and DKK3 has been reported to induce changes of β‐catenin localization in cervical cancer 27. Thus, to reveal this question in pancreatic cancer, we examined the changes of β‐catenin localization induced by hypoxia in NC and DKK3 transfectants by indirect immunofluorescence detection of the protein in Bxpc‐3 cells.

**Figure 2:**
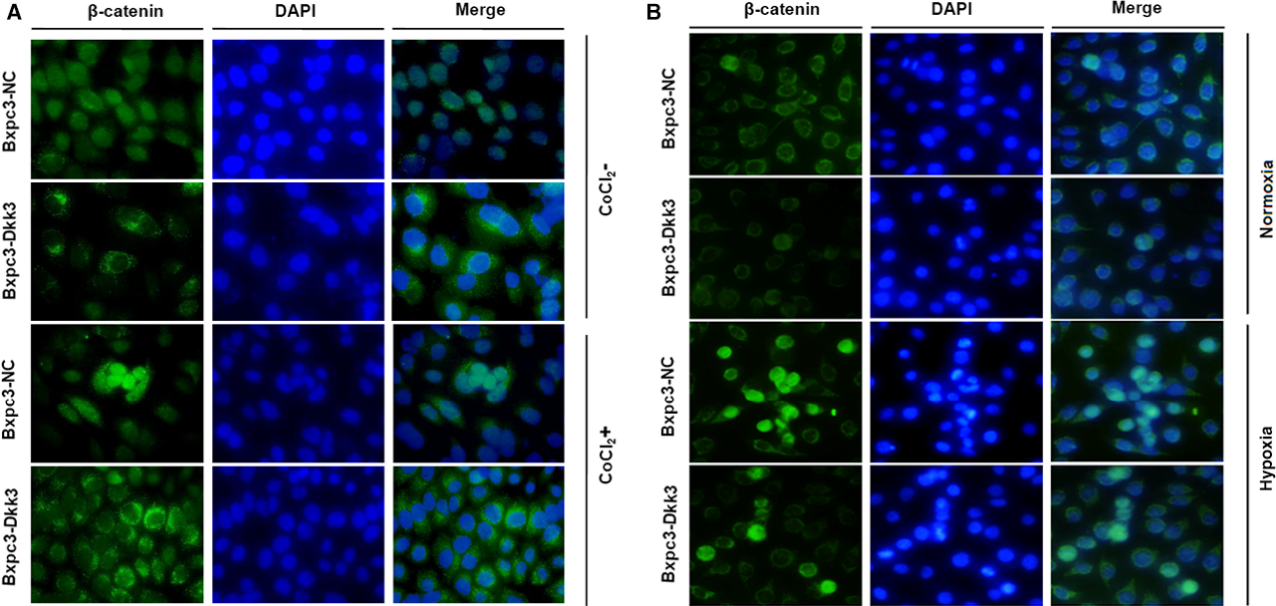
Immunofluorescence images of nuclear translocation of β‐catenin in Bxpc‐3 cells with Dickkopf‐related protein 3 (DKK3) overexpression and their control cells in hypoxia mimetic conditions (**A**) and hypoxic conditions (**B**).

**Figure 3:**
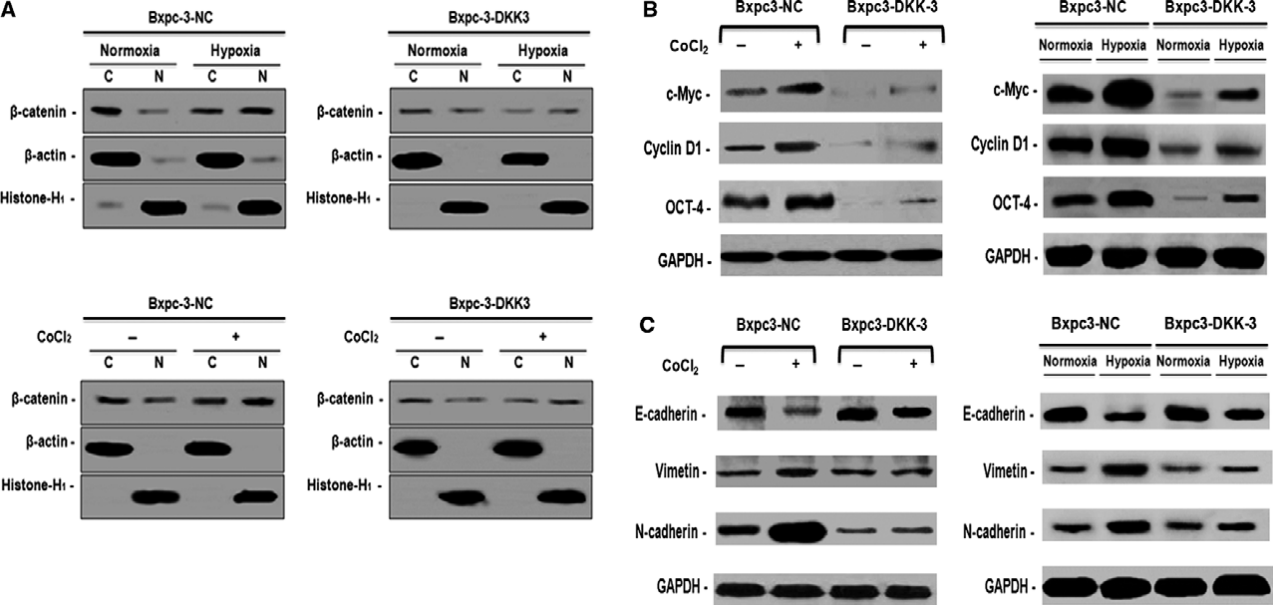
Ectopic expression of Dickkopf‐related protein 3 (DKK3) disrupted β‐catenin/TCF‐4 signalling and suppresses epithelial–mesenchymal transition in pancreatic cancer Bxpc‐3 cell in hypoxic conditions. (**A**) Western blotting analysis of cytoplasm and nuclear levels of β‐catenin in hypoxic conditions and hypoxia mimetic conditions. (**B**) Western blotting analysis of β‐catenin downstream targets gene and stem cell marker Oct‐4 in hypoxic conditions and hypoxia mimetic conditions. (**C**) Western blotting analysis of epithelial‐mesenchymal transition (EMT) markers in hypoxic conditions and hypoxia mimetic conditions.

As shown in Figure 2A and B, β‐catenin is activated and normally localized in the cytoplasm and nucleus in pancreatic cancer cell. However, whether in hypoxic conditions or hypoxia mimetic conditions for 48 hrs_,_ activated β‐catenin translocated to the nucleus significantly increased. Followed by DKK3 transfection, β‐catenin showed predominant cytoplasmic localization in a significant fraction of cells, whether hypoxia or not. Similarly, immunoblotting showed DKK3 decreased the elevated levels of nuclear β‐catenin induced by hypoxia in pancreatic cancer cells, supporting the result of immunofluorescence (Fig. 3A). Furthermore, the expression of c‐Myc and cyclin D1, two well known TCF‐4 downstream targets 13, was significantly suppressed by DKK3 (Fig. 3B). These results suggested that DKK3 inhibited β‐catenin/TCF‐4 signalling by preventing the nuclear translocation of β‐catenin in pancreatic cancer Bxpc‐3 cell.

### DKK3 suppresses epithelial–mesenchymal transition and migration of pancreatic cancer Bxpc‐3 cell in hypoxic conditions

Epithelial–mesenchymal transition is one of the mechanisms for cancer cells to acquire chemoresistance including pancreatic cancer. β‐catenin signals is recognized as one of the cell signalling pathways involved in EMT 28. Next, we suggested weather DKK3 regulated EMT in pancreatic cancer by suppressed β‐catenin signalling in hypoxic conditions. Western blot analysis showed up‐regulated pancreatic cancer mesenchymal marker vimentin and N‐cadherin and down‐regulated epithelial marker E‐cadherin in hypoxic conditions. However, in DKK3‐expressing cells, the phenomenon was reversed (Fig. 3C), indicating that DKK3 indeed negatively regulates EMT.

**Figure 4:**
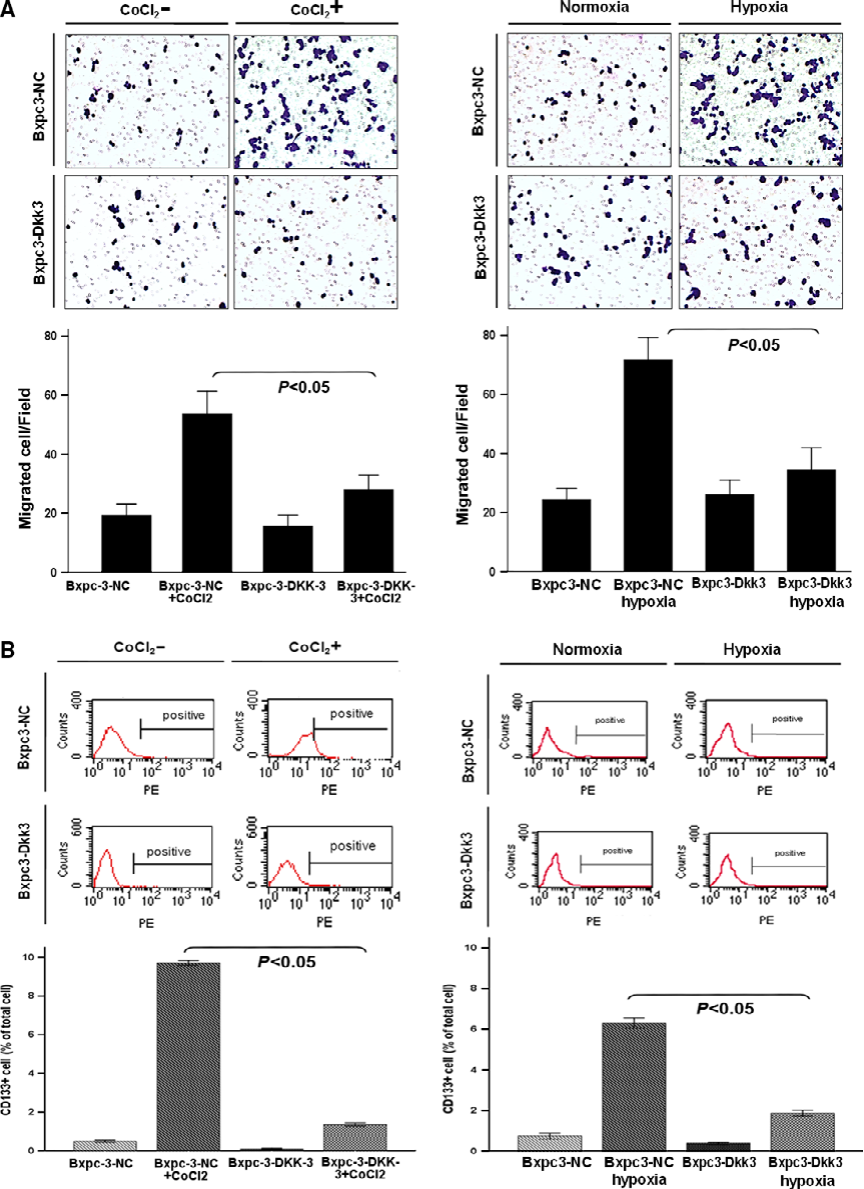
Ectopic expression of Dickkopf‐related protein 3 (DKK3) suppress cell migration and stem cell of pancreatic cancer cell. (**A**) The cell migration of vector‐ or DKK3‐transfected cells was tested by transwell assay in hypoxic conditions and hypoxia mimetic conditions. All of the experiments were performed in triplicate. (**B**) CD133^+^ pancreatic cancer cells were analysed by flow cytometry in vector‐ or DKK3‐transfected cells in hypoxic conditions and hypoxia mimetic conditions. All of the experiments were performed in triplicate.

Transwell assay was further performed to uncover the effect of DKK3 on cell migration. Results showed that the number of migrated vector‐transfected cells in hypoxic conditions remarkably increased compared with in normal environment. However, the number of migrated cells in DKK3‐expressing group was significantly less than the normal control group in hypoxic conditions (Fig. 4A), indicating that DKK3 inhibits cell migration induced by hypoxia.

As EMT is implicated in regulating stem cell properties, we further investigated the expression of stem cell‐associated markers in DKK3‐transfected pancreatic cancer cells. As showed in Figure 3B, relative to normal control group, DKK3 down‐regulated stem markers OCT4 whether in hypoxic conditions or not. To further verify the effect of DKK3 on stem cell, CD133^+^ pancreatic cancer cells were analysed by flow cytometry. At hypoxic conditions and hypoxia mimetic conditions, relative to normal conditions, vector‐transfected cells were induced much more CD133^+^ pancreatic cancer cells (6.33% *versus* 0.70%, 9.71% *versus* 0.48%, respectively). But, in DKK3‐transfected pancreatic cancer Bxpc‐3 cell, the percentage of CD133^+^ cells significantly fell to 1.35, 0.09 and 1.19, 0.36 respectively. It is suggesting that DKK3 may reverse the stem cell‐like phenotype of tumour cells in hypoxic conditions (Fig. 4B).

### DKK3 sensitizes pancreatic cancer Bxpc‐3 cell to gemcitabine

**Figure 5:**
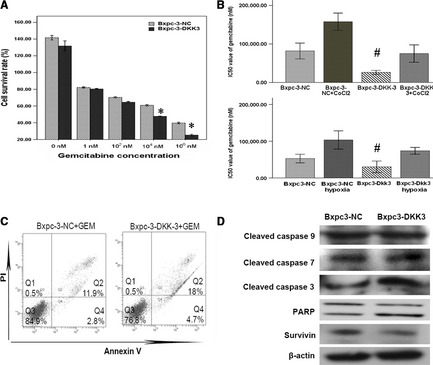
Dickkopf‐related protein 3 (DKK3) induced proliferation inhibition and apoptosis in pancreatic cancer Bxpc‐3 cell. (**A**) Vector‐transfected and DKK3‐transfected cells were treated with increasing concentrations of gemcitabine (0, 10, 10^2^, 10^4^, 10^6^ nM) for 72 hrs. *P < 0.05, by comparison with Bxpc3‐NC group. (**B**) IC50 value of gemcitabine treatment was measured in hypoxic conditions and hypoxia mimetic conditions. ^#^ *P* < 0.05, by comparison with Bxpc3‐NC group. (**C**) Apoptosis rate analysis using Annexin V/propidium iodide flow cytometry in vector‐transfected and DKK3‐transfected cells treated with gemcitabine. (**D**) Western blotting analysis of apoptosis‐related protein in vector‐transfected and DKK3‐transfected cells after gemcitabine treatment.

To further investigate the utility of DKK3 in gemcitabine treatment of pancreatic cancer Bxpc‐3 cell, CCK‐8 assay was performed. For these studies, vector‐transfected and DKK3‐transfected cells were treated with increasing concentrations of gemcitabine (0, 10, 10^2^, 10^4^, 10^6^ nM) for 72 hrs. In 0–10^2^ nM gemcitabine, no significant change in the cell survival rate was observed between the vector‐transfected and DKK3‐transfected cells. However, in 10^4^ and 10^6^ nM gemcitabine, the cell survival rate was 60.9, 39.7% and 45.7, 25.3% in the vector and DKK3‐transfected cells respectively (Fig. 5A). The IC_50_ value of gemcitabine treatment for 72 hrs was higher (*P* < 0.05) in the vector cells than DKK3‐transfected cells in normal oxygen environment (Fig. 5B). In addition, to determine whether DKK3 enhances the induction of apoptosis by gemcitabine, flow cytometry analysis of apoptosis was performed. We found that cells with DKK3 expression underwent significant apoptosis compared to controls, with 22.7% of cells staining positive for Annexin V (Fig. 5C). To evaluate the mechanism of DKK3 in inducing cell apoptosis, we thus investigated apoptosis‐related protein by Western blotting. Western blotting analysis showed up‐regulated the cleavage of PARP and caspase‐3 and down‐regulated survivin in DKK3‐expressing cells (Fig. 5D). However, no change in the expression of cleaved caspase‐7, 9 were found in Bxpc‐3 cells with DKK3 expression.

### DKK3 delays tumour growth and augments gemcitabine therapeutic effect in pancreatic cancer xenotransplantation model

**Figure 6:**
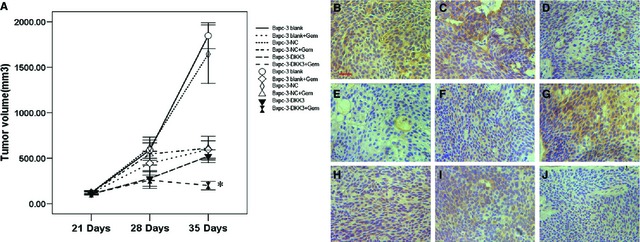
The effect of overexpression of Dickkopf‐related protein 3 (DKK3) on tumour growth *in vivo*. (**A**) Measurements of tumour volume at various time‐points revealing *in vivo* therapeutic efficacy of gemcitabine. DKK3 significantly decreased the mean tumour volume compared to normal control and blank group animals (*P < 0.05; *n* = 6). Points, mean; bars, S.E. Immunohistochemical analysis of β‐catenin (**B**–**D**), E‐cadherin (**E**–**G**) and CD133 (**H**–**J**) protein expression in pancreatic subcutaneous tumour samples from blank, NC and DKK3 group, respectively, scale bar: 50 μm.

To examine the effect of overexpression of DKK3 *in vivo*, Bxpc‐3‐DKK3, Bxpc‐3‐NC and Bxpc‐3 cells were implanted into the athymic mice. Xenografts were established in athymic mice within 3 weeks and subjected to treatment with vehicle (0.9% sodium chloride), gemcitabine (125 mg/kg) twice per week for 2 weeks. At the end of treatment, on day 14 after the start of treatment, tumour volumes showed significant decrease in the Bxpc‐3‐DKK3 group compared with Bxpc‐3‐NC and Bxpc‐3 (*P* < 0.05; Fig. 6A). Furthermore, the expression of CD133 (Fig. 6H–J) and β‐catenin (Fig. 6B–D) were significantly down‐regulated and E‐cadherin (Fig. 6E–G) was remarkably up‐regulated in the DKK3 group compared to the normal control and blank group.

## Discussion

Wnt/β‐catenin signalling is frequently activated in many cancers, and plays an important role in tumour initiation and progression. It has been shown that the expression of Wnt antagonists are often down‐regulated in several human cancers and DKK3 was no exception 29. There are reports on DKK3 both enhancing and repressing the Wnt signalling pathway 30, 31. Recently, DKK3 was also reported as playing distinct roles in different human pancreatic cancer cells, but not much is known about the detailed mechanism 20, 32.

In this study, we detected DKK3 protein expression in human pancreatic cancer cells. We found that DKK3 expression was significantly higher in MiaPaCa‐2, PANC‐1, SW1900 cells and DKK3 was not expressed in Aspc‐1, Bxpc‐3 and CFPAC‐1 cells. The results are partially in agreement with those of article 32, 33. However, there are no differences with Zenzmaier *et al*. 20. DKK3 down‐regulation and its transcriptional silencing is at least partly because of aberrant hypermethylation of the promoter 34. In this study, we demonstrated that DKK3 methylation was found in the pancreatic cancer cell lines, in which DKK3 was silenced. The result was partially consistent with previous studies of DKK3 down‐regulation and methylation in pancreatic cancer cell lines 32. Dickkopf‐related protein 3 expression in pancreatic cancer specimen remains unknown. Furthermore, IHC analysis to look at the DKK3 expression level in pancreatic cancer tissues was performed. Our study demonstrated that DKK3 expression was significantly lower in pancreatic cancer tissues, a finding consistent with the previous study 35, which has been reported for renal cancer tissues.

Hypoxia within tumours causes dysfunction of the E‐cadherin/β‐catenin complex with an accumulation of β‐catenin in the nucleus and produces an invasive phenotype of tumour cells 36. Severe intratumoural hypoxia is present in pancreatic cancer 25, 37, suggesting that hypoxia plays a role in the accumulation and activation of β‐catenin in pancreatic cancer cells. As a Wnt antagonists, it is reported that DKK3 overexpression does not affect nuclear/cytoplasmic accumulation of β‐catenin 11. On the other hand, another article has shown that overexpression of DKK3 leads to a reduction in the cytoplasmic level of β‐catenin in SaOS‐2 cells 31. In a previous report, Xiang *et al*. have reported that DKK3 inhibited the activation of β‐catenin and its downstream genes by abrogating its nuclear translocalization in breast cancer 34. Thus, we examined the function of DKK3 on β‐catenin in hypoxia in pancreatic cancer Bxpc‐3 cell using DKK3‐overexpressing transfectants. In the results, we revealed that hypoxia conditions or hypoxia mimetic conditions really induced β‐catenin nucleus translocation and the transcriptional activation of β‐catenin in Bxpc‐3 cell. However, after DKK3 transfection, consistent with the results reported by Xiang, we also found that DKK3 inhibits the nuclear accumulation and the activation of β‐catenin and its downstream genes cyclin D1, c‐Myc in pancreatic cancer Bxpc‐3 cell in normoxia and hypoxia.

Epithelial–mesenchymal transition enables epithelial cells to acquire motility and invasiveness that are characteristic of mesenchymal cells 38. It plays an important role in development and cancer cell metastasis, drug resistance including pancreatic cancer 7, 38, 39. One of the signals initiating an EMT is nuclear translocation of β‐catenin induced by hypoxia 6, 40 and EMT is implicated in regulating stem cell properties in hypoxic conditions 8, 41. In addition, pancreatic cancer cells might acquire stemness or express ‘stem cell‐like’ properties under hypoxia, consequently leading to an aggressive phenotype 42. A previous study confirmed that hypoxia induced an increased number of invading CD133^+^ pancreatic cancer cells 42, a marker typically used to identify and isolate human pancreatic cancer stem cells 43. Thus, we wondered whether DKK3 has an effect on EMT and stem cell properties in pancreatic cancer. In pancreatic cancer Bxpc‐3 cell, the overexpression of DKK3 was shown to suppress EMT and migration of cell in hypoxic conditions. We also further demonstrated DKK3 down‐regulated stem cell markers OCT4 and induced the decreased number of CD133^+^ pancreatic cancer Bxpc‐3 cell whether under hypoxia or not.

Gemcitabine is the only approved drug for the treatment of pancreatic cancer 44. Dickkopf‐related protein 3 has been reported to inhibit cancer proliferation and induce apoptosis in several cancers 35, 45. Therefore, we investigated the effect of DKK3 on proliferation and apoptosis of pancreatic cancer Bxpc‐3 cell treated by gemcitabine. We found that cell proliferation was significantly inhibited by reducing the IC_50_ value of gemcitabine and the apoptotic cell number was increased after gemcitabine treatment in DKK3 transfection. To probe the mechanism of DKK3 on apoptosis, Western analysis of several apoptosis‐related genes was performed. Among them, the protein expression of cleaved caspase‐3 and PARP was significantly increased in DKK3‐transfected cell, accompanied with survivin decreasing. It has been reported that cleaved caspase‐3 was involved in the DKK3‐induced apoptotic pathway 46, and that survivin has been described as a β‐catenin/Tcf/Lef target gene 47. Thus, our results are consistent with these previous reports.

In addition to these *in vitro* results, we found that the tumour size of DKK3 transfectants in nude mice was significantly decreased compared to control cells. We also observed DKK3 potentiates the antitumour effects of gemcitabine in a subcutaneous xenograft pancreatic cancer. Most importantly, the results *in vitro* were replicated *in vivo*, not only down‐regulating the expression of CD133 and β‐catenin but also increasing the expression of E‐cadherin in tumour tissues transfected with DKK3.

In conclusion, these experiments demonstrated that DKK3 suppresses EMT of pancreatic cancer Bxpc‐3 cell in hypoxic conditions by blocking the translocation of β‐catenin to nucleus, leading to the enhancing of the antitumour effects of gemcitabine in pancreatic cancer Bxpc‐3 cell. These results indicate DKK3 may be a novel target for treatment against the drug‐resistant in DKK3 negative pancreatic cancer. However, further investigations are necessary to probe the detail mechanism of DKK3 in treatment of pancreatic cancer.

## Conflicts of interest

The authors confirm that there are no conflicts of interest.
